# International Analysis of Age-Specific Mortality Rates From Mesothelioma on the Basis of the International Classification of Diseases, 10th Revision

**DOI:** 10.1200/JGO.2017.010116

**Published:** 2017-08-11

**Authors:** Paolo Boffetta, Matteo Malvezzi, Enrico Pira, Eva Negri, Carlo La Vecchia

**Affiliations:** **Paolo Boffetta**, Icahn School of Medicine at Mount Sinai, New York, NY; **Matteo Malvezzi**, **Eva Negri**, and **Carlo La Vecchia**, University of Milan, Milan; and **Enrico Pira**, University of Turin, Turin, Italy.

## Abstract

Past analyses of mortality data from mesothelioma relied on unspecific codes, such as pleural neoplasms. We calculated temporal trends in age-specific mortality rates in Canada, the United States, Japan, France, Germany, Italy, the Netherlands, Poland, the United Kingdom, and Australia on the basis of the 10th version of the International Classification of Diseases, which includes a specific code for mesothelioma. Older age groups showed an increase (in the United States, a weaker decrease) during the study period, whereas in young age groups, there was a decrease (in Poland, a weaker increase, starting, however, from low rates). Results were consistent between men and women and between pleural and peritoneal mesothelioma, although a smaller number of events in women and for peritoneal mesothelioma resulted in less precise results. The results show the heterogeneous effect of the reduction of asbestos exposure on different age groups; decreasing mortality in young people reflects reduced exposure opportunity, and increasing mortality in the elderly shows the long-term effect of early exposures.

Descriptive cancer epidemiology relies on data on cancer incidence, typically from population-based cancer registries, and cancer mortality, typically from national statistics.^1^ Data from cancer registries are of better quality because they include histologic verification of most of the patients; however, in the case of rare neoplasms, they are limited by the relatively small size of many cancer registries. Mortality data rely on medical certification in most countries, whose diagnostic accuracy is suboptimal.^2^

Because the occurrence of mesothelioma is relatively rare in most populations, its descriptive epidemiology is largely based on mortality data,^3^ which are coded on the basis of subsequent versions of the International Classification of Diseases (ICD).^4^ Until the 10th revision of the ICD (ICD10), however, mesothelioma was not associated with a specific code, and mesothelioma deaths were classified under neoplasms of the pleura (ICD, 9th revision [ICD9] code 163), neoplasms of the peritoneum (ICD9 code 158), and under other organs, such as the pericardium and the tunica vaginalis, where mesothelioma rarely occurs. Other tumor types were also included in these rubrics, complicating the use of mortality data to describe geographic and temporal patterns of the disease.

In ICD10,^5^ a specific code was introduced for mesothelioma (from any site), which enables more valid analyses of mortality data from populations in which the new classification has been adopted; this occurred in the late 1990s and early 2000s in many high-income countries. Because mortality data on the basis of ICD10 have become available for a period of ≥ 15 years in several countries, we aimed to analyze international temporal patterns of age-adjusted and age-specific trends in mesothelioma mortality.

The WHO database (WHO Statistical Information System) provides official death certification data for most cancer sites; we considered those for mesothelioma from the first year of use of the ICD10 until the most recent year. We restricted the analysis to selected high-income countries providing valid and consistent data on mesothelioma and at least 15 million inhabitants (Canada [2000-2011], the United States [1999-2014], Japan [1995-2013], France [2000-2013], Germany [1998-2014], Italy [2003-2012], the Netherlands [1996-2013], Poland [1999-2014], the United Kingdom [2001-2013], and Australia [1998-2014]). We considered deaths from all mesothelioma (ICD10 C45), as well as from pleural mesothelioma (ICD10 C45.0) and peritoneal mesothelioma (ICD10 C45.1). In the latter analysis, we attributed deaths from mesothelioma from unspecified sites (ICD10 C45.9) to the pleura (85% in men, 73% in women) and peritoneum (7% in men, 18% in women) on the basis of the distribution of the patients registered in the SEER program during 2003 to 2008.^6^

We computed age-specific mortality rates for each 5-year age group (from 0-4 to ≥ 85 years) for each year and for the periods 2000 to 2004, 2005 to 2009, and 2013 (or closest available year, 2011 in Canada, and 2012 in Italy). We calculated age-standardized rates (world standard population) per 100,000 men and women, at all ages, using the direct method, as well as for the age groups 35 to 54, 55 to 64, 65 to 74 and ≥ 75 years.^7^ We also fit a logarithmic Poisson count data joinpoint regression model to identify trend changes for all ages and each age group.^8^

Mortality rates for all mesotheliomas are reported in Figure 1; average annual percent changes are listed in Table 1; overall age-standardized rates in 2000 to 2004 and in 2013 are reported in Appendix Table A1, and detailed results of the joinpoint analysis are reported in Appendix Table A2. In 2000 to 2004, rates in men were > 2 of 100,000 in the Netherlands, United Kingdom, and Australia; between 1.0 and 1.3 of 100,000 in France, Germany, and Italy; approximately 0.7 of 100,000 in Canada and the United States; and < 0.5 of 100,000 in Japan and Poland. In 2013, overall rates tended to decrease in the Netherlands, Australia, the United States, and France (and to a small extent, in the United Kingdom) and tended to increase in Japan and mostly in Poland (to reach 0.6 of 100,000). Overall female rates were lower, between 0.1 and 0.5 of 100,000, the highest one in 2000 to 2004 being in Italy, the United Kingdom, and Australia. No appreciable change was observed between 2000 to 2004 and 2013, except in Poland, whose rates rose from 0.07 to 0.21 of 100,000; a small increase was apparent in the United Kingdom and Australia as well.

**Table 1:**
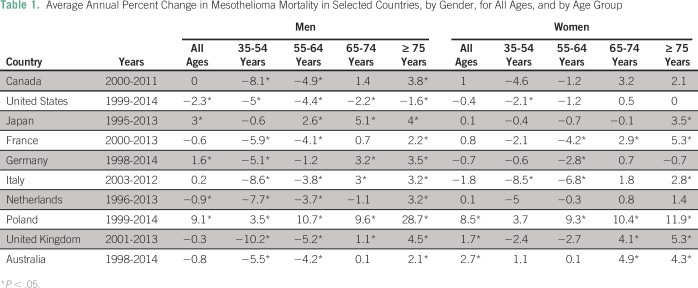
Average Annual Percent Change in Mesothelioma Mortality in Selected Countries, by Gender, for All Ages, and by Age Group

**Fig 1:**
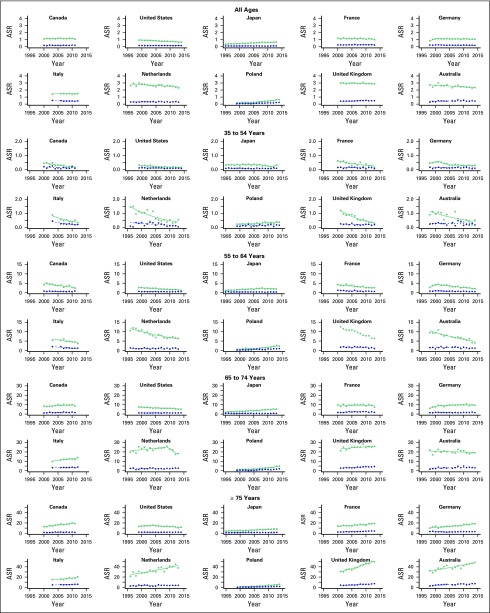
Mesothelioma mortality rates on the basis of International Classification of Diseases, 10th Revision, by gender, in selected countries, for all ages, and by age group. ASR, age-standardized rate per 100,000. Men, squares; women, circles.

The analysis by age group among men showed a consistent pattern in most countries in the analysis: older age groups showed an increase (in the United States, a weaker decrease) in mortality rates during the period of study, whereas in young age groups, there was a decrease (in Poland, a weaker increase). The magnitude of the increase (in older age groups) or the decrease (in younger age groups) varied across countries, as it varied the years in which changes in trends were identified by the joinpoint analysis.

The results of the age-specific analysis among women revealed a pattern similar to that identified among men, with mortality trends being negative (or less positive) in the young and positive (or less negative) in the elderly, although in some of the countries (eg, Germany), this shift from negative across age groups was not monotonic, and the absolute value of the change varied across countries.

We repeated the analysis separating pleural and peritoneal mesothelioma (Appendix Figs A1 and A2; Appendix Table A3). Because pleural mesothelioma deaths represented the vast majority of the total, trends and patterns for this form of the disease paralleled those of the main analysis. The assessment of trends of peritoneal mesothelioma among patients younger than 55 years of age was hampered by a small number of deaths; in the other age groups, however, patterns were similar to those observed for all forms of the disease.

The analysis of trends in mortality from mesothelioma on the basis of ICD10 showed variability in the absolute levels and in the presence and magnitude of an increasing (or decreasing) trend. Despite this heterogeneity, a consistent pattern was shown in that mesothelioma rates were decreasing among younger people, whereas they were still increasing among older people. The only countries with a different pattern were the United States (decrease in all age groups among men) and Poland (increase in all age group and both sexes).

The trends in age-adjusted rates are consistent with those reported in recent years for individual countries on the basis of either mortality or incidence data, for example, Australia,^9^ Germany,^10^ the United States,^6^ and England.^11^ An analysis of temporal trends in age-specific rates, however, was reported only for 1998 to 2002 in southeast England^12^; its results were similar to ours, although on the basis of small numbers.

The decreases observed in the United States as contrasted with western Europe were already observed in an analysis of trends between 1973 and 2003^13^ and in an age-period-cohort analysis of trends until the end of last century,^14^ and are attributable to earlier control of asbestos (mainly amphibole) exposure in the United States than in other high-income countries. Japan had relatively low rates in middle-aged and elderly people in the early 2000s, but showed appreciable increases over the calendar period considered, reflecting changes in asbestos imports in the past in that country.^15^ Poland started from extremely low rates, which were probably real, because the validity of Polish cancer death certification has long been acceptable.^16^

Mesothelioma mortality rates were low in Eastern compared with Western Europe,^14^ but show a tendency toward leveling or even overcoming western European rates in younger generations. This likely reflects the changing pattern and type of asbestos exposure in this region of the world.

Given the strong relationship between asbestos exposure (mainly at the workplace) and occurrence of mesothelioma,^17^ and the fact that the prevalence of occupational exposure has declined in the last decades because of stricter regulations on asbestos use,^18^ it is plausible that the results of our analysis represent the effect of reduction of asbestos exposure in the younger age groups. The decline in mortality rates among young people shows the benefit of reduced opportunity to experience occupational exposure throughout the working life; in fact, these people were born approximately in the 1950s and started their working life in the 1970s and 1980s, when restrictive asbestos regulations were implemented. However, trends in the young age groups should be interpreted with caution because of the small number of deaths in this category. People ages 55 to 74 years, however, had a higher probability of exposure during the early part of their working history, whereas those ≥ 75 years of age experienced the full extent of the epidemic of asbestos exposure, at least during the first part of their employment experience. This interpretation is consistent with a predominant role of early exposure to asbestos in determining subsequent risk of mesothelioma and with a modest role of subsequent quitting or continuing exposure.^19^ These results also show the powerful effect of measures aimed at preventing asbestos exposure, which have been implemented during the last decades: 2013 mortality rates in men ages 35 to 54 years were, in most countries, in the range 0.15 to 0.30 of 100,000, a level well below those measured a decade earlier, and the decline is likely to continue in the coming years.

## Figures and Tables

**Fig A1:**
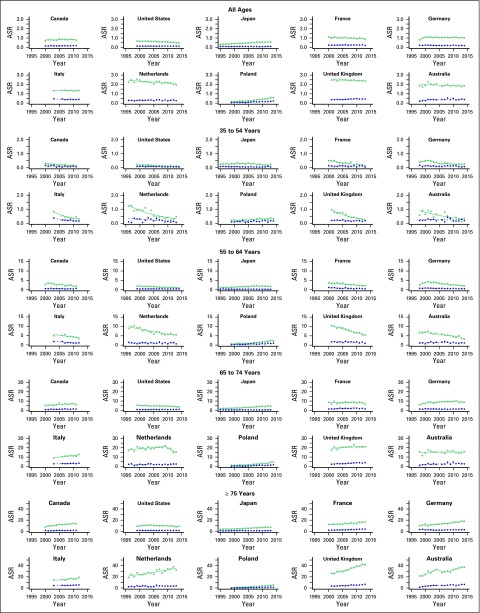
Pleural mesothelioma mortality rates on the basis of the International Classification of Diseases (10th revision) codes by gender in selected countries, by all ages, and by age group. ASR, age-standardized rate per 100,000. Men, squares; women, circles.

**Fig A2:**
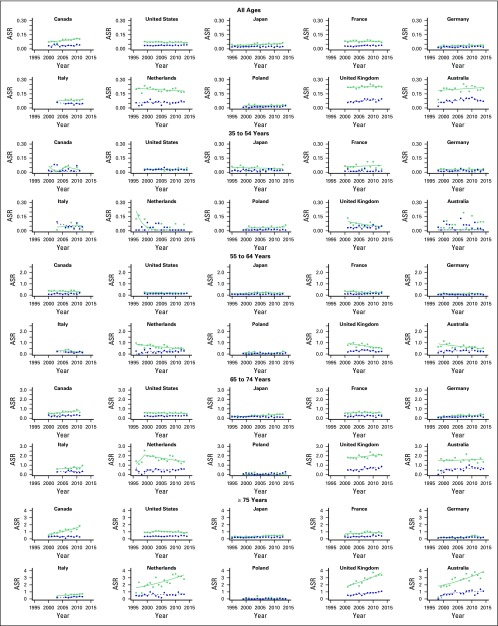
Peritoneal mesothelioma mortality rates on the basis of the International Classification of Diseases (10th revision) codes by gender in selected countries, by all ages, and by age group. ASR, age-standardized rate per 100,000. Men, squares; women, circles.

**Table A1:**
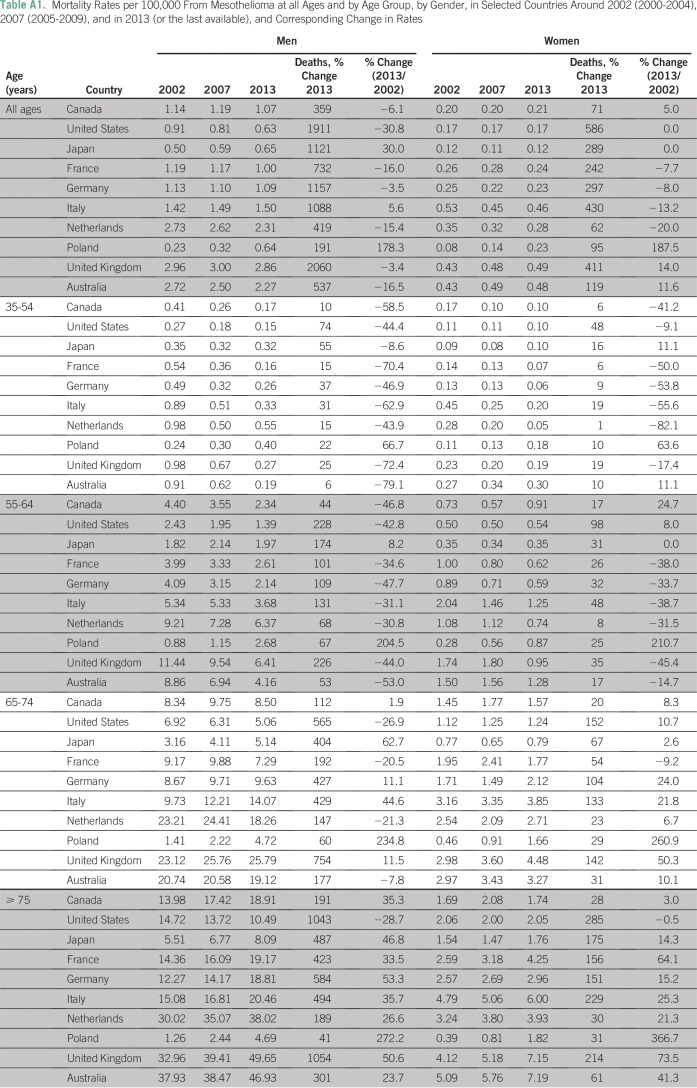
Mortality Rates per 100,000 From Mesothelioma at all Ages and by Age Group, by Gender, in Selected Countries Around 2002 (2000-2004), 2007 (2005-2009), and in 2013 (or the last available), and Corresponding Change in Rates

**Table A2:**
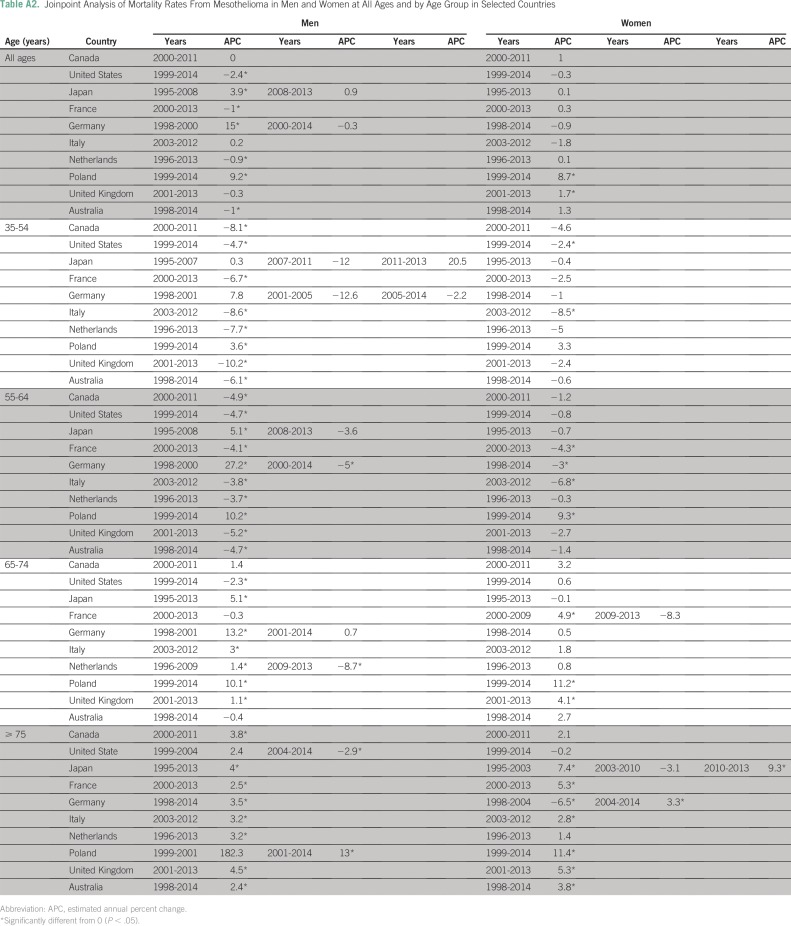
Joinpoint Analysis of Mortality Rates From Mesothelioma in Men and Women at All Ages and by Age Group in Selected Countries

**Table A3:**
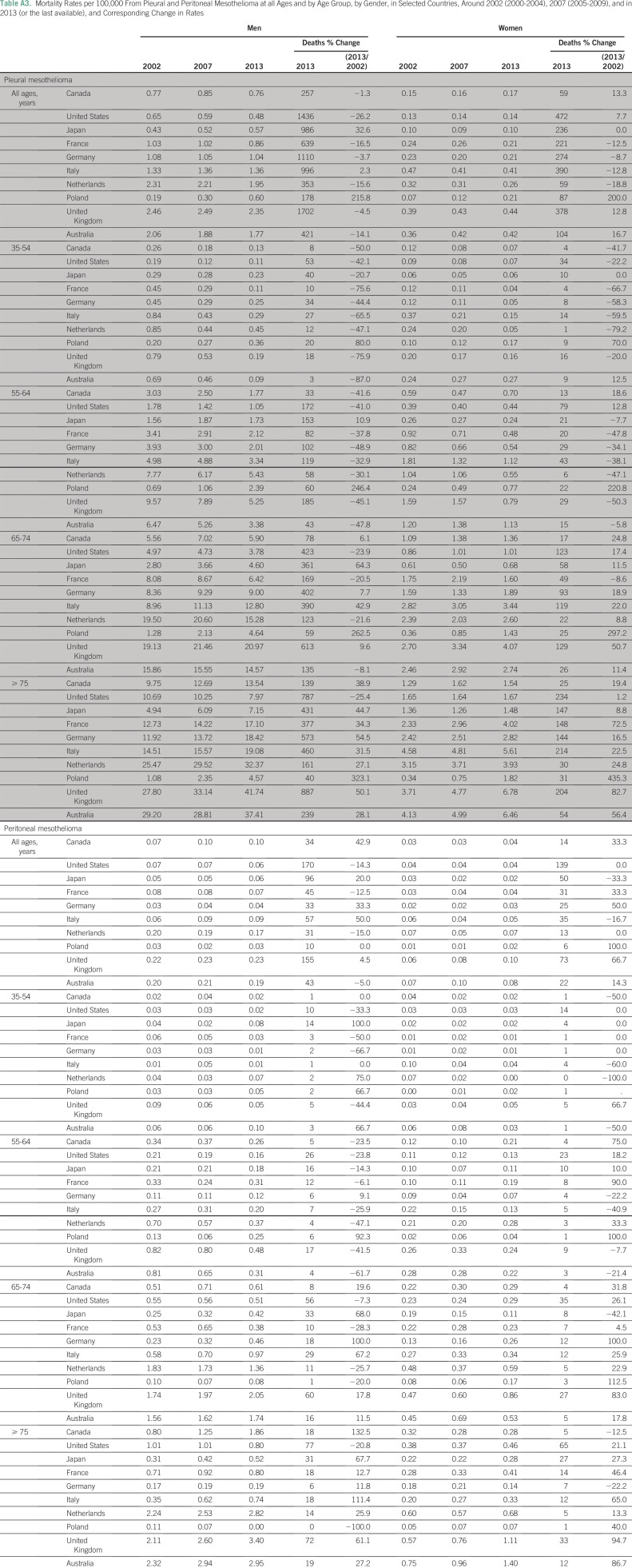
Mortality Rates per 100,000 From Pleural and Peritoneal Mesothelioma at all Ages and by Age Group, by Gender, in Selected Countries, Around 2002 (2000-2004), 2007 (2005-2009), and in 2013 (or the last available), and Corresponding Change in Rates
